# Potential anti-COVID-19 agents, cepharanthine and nelfinavir, and their usage for combination treatment

**DOI:** 10.1016/j.isci.2021.102367

**Published:** 2021-03-26

**Authors:** Hirofumi Ohashi, Koichi Watashi, Wakana Saso, Kaho Shionoya, Shoya Iwanami, Takatsugu Hirokawa, Tsuyoshi Shirai, Shigehiko Kanaya, Yusuke Ito, Kwang Su Kim, Takao Nomura, Tateki Suzuki, Kazane Nishioka, Shuji Ando, Keisuke Ejima, Yoshiki Koizumi, Tomohiro Tanaka, Shin Aoki, Kouji Kuramochi, Tadaki Suzuki, Takao Hashiguchi, Katsumi Maenaka, Tetsuro Matano, Masamichi Muramatsu, Masayuki Saijo, Kazuyuki Aihara, Shingo Iwami, Makoto Takeda, Jane A. McKeating, Takaji Wakita

**Affiliations:** 1 Department of Virology II, National Institute of Infectious Diseases, Tokyo 162-8640, Japan; 2 Department of Applied Biological Science, Tokyo University of Science, Noda 278-8510, Japan; 3 Institute for Frontier Life and Medical Sciences, Kyoto University, Kyoto 606-8507, Japan; 4 MIRAI, JST, Saitama 332-0012, Japan; 5 The Institute of Medical Science, The University of Tokyo, Tokyo 108-8639, Japan; 6 AIDS Research Center, National Institute of Infectious Diseases, Tokyo 162-8640, Japan; 7 Department of Biology, Faculty of Sciences, Kyushu University, Fukuoka 812-8581, Japan; 8 Cellular and Molecular Biotechnology Research Institute, National Institute of Advanced Industrial Science and Technology, Tokyo 135-0064, Japan; 9 Division of Biomedical Science, Faculty of Medicine, University of Tsukuba, Tsukuba 305-8575, Japan; 10 Transborder Medical Research Center, University of Tsukuba, Tsukuba 305-8575, Japan; 11 Faculty of Bioscience, Nagahama Institute of Bio-Science and Technology, Nagahama 526-0829, Japan; 12 Graduate School of Science and Technology, Nara Institute of Science and Technology, Ikoma 630-0192, Japan; 13 Center for Research and Education on Drug Discovery, Faculty of Pharmaceutical Sciences, Hokkaido University, Sapporo 060-0812, Japan; 14 Department of Virology, Faculty of Medicine, Kyushu University, Fukuoka 812-8582, Japan; 15 Department of Virology I, National Institute of Infectious Diseases, Tokyo 162-8640, Japan; 16 Department of Epidemiology and Biostatistics, Indiana University School of Public Health-Bloomington, Bloomington, IN 47405, USA; 17 National Center for Global Health and Medicine, Tokyo 162-8655, Japan; 18 Faculty of Pharmaceutical Sciences, Tokyo University of Science, Noda 278-8510, Japan; 19 Research Institute for Science and Technology, Tokyo University of Science, Noda 278-8510, Japan; 20 Department of Pathology, National Institute of Infectious Diseases, Tokyo 162-8640, Japan; 21 Laboratory of Biomolecular Science, Faculty of Pharmaceutical Sciences, Hokkaido University, Sapporo 060-0812, Japan; 22 Global Station for Biosurfaces and Drug Discovery, Center for Life Innovation, Hokkaido University, Sapporo 060-0812, Japan; 23 International Research Center for Neurointelligence, The University of Tokyo Institutes for Advanced Study, The University of Tokyo, Tokyo 113-8654, Japan; 24 Institute for the Advanced Study of Human Biology (ASHBi), Kyoto University, Kyoto 606-8501, Japan; 25 NEXT-Ganken Program, Japanese Foundation for Cancer Research (JFCR), Tokyo 135-8550, Japan; 26 Science Groove Inc., Fukuoka 810-0041, Japan; 27 Department of Virology III, National Institute of Infectious Diseases, Tokyo 208-0011, Japan; 28 Nuffield Department of Medicine, University of Oxford, Oxford OX3 7FZ, UK; 29 Research Center for Drug and Vaccine Development, National Institute of Infectious Diseases, Tokyo 162-8640, Japan

**Keywords:** Medical Substance, Pharmaceutical Preparation, Virology

## Abstract

Antiviral treatments targeting the coronavirus disease 2019 are urgently required. We screened a panel of already approved drugs in a cell culture model of severe acute respiratory syndrome coronavirus 2 (SARS-CoV-2) and identified two new agents having higher antiviral potentials than the drug candidates such as remdesivir and chroloquine in VeroE6/TMPRSS2 cells: the anti-inflammatory drug cepharanthine and human immunodeficiency virus protease inhibitor nelfinavir. Cepharanthine inhibited SARS-CoV-2 entry through the blocking of viral binding to target cells, while nelfinavir suppressed viral replication partly by protease inhibition. Consistent with their different modes of action, synergistic effect of this combined treatment to limit SARS-CoV-2 proliferation was highlighted. Mathematical modeling *in vitro* antiviral activity coupled with the calculated total drug concentrations in the lung predicts that nelfinavir will shorten the period until viral clearance by 4.9 days and the combining cepharanthine/nelfinavir enhanced their predicted efficacy. These results warrant further evaluation of the potential anti-SARS-CoV-2 activity of cepharanthine and nelfinavir.

## Highlights

• Nelfinavir (NFV) inhibits SARS-CoV-2 replication
• Cepharanthine (CEP) inhibits SARS-CoV-2 attachment to target cells
• Combination of NFV and CEP can synergistically augment antiviral activity
• NFV and CEP are estimated to shorten the time to viral elimination at clinical doses

## Graphical Abstract

**Figure:**
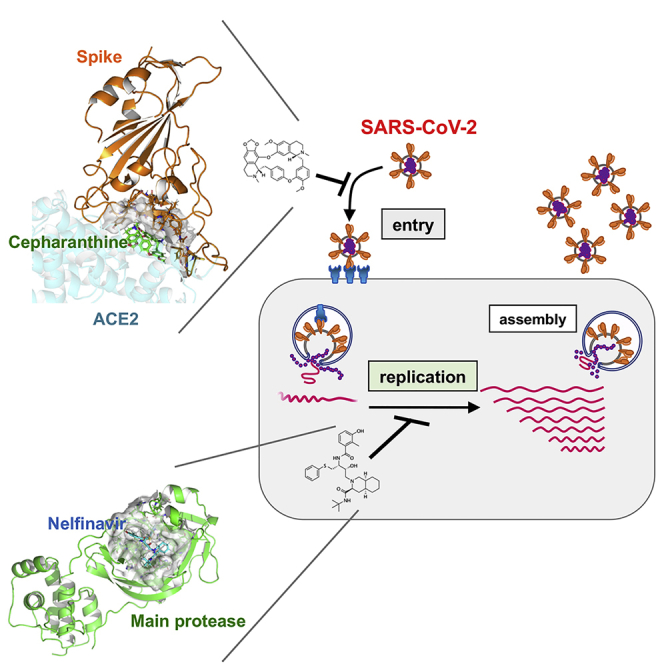

## Introduction

The novel coronavirus disease 2019 (COVID-19), caused by the infection of severe acute respiratory syndrome coronavirus 2 (SARS-CoV-2), is a global public health problem that is impacting social and economic damage worldwide (Huang et al., 2020; Zhou et al., 2020; Zhu et al., 2020). More than 5,000,000 confirmed cases with over 300,000 deaths were reported late May 2020 across 216 countries/areas/territories (WHO, 2020). COVID-19 was characterized as a pandemic by the World Health Organization (WHO), and new treatments along with a vaccine are urgently needed. Remdesivir (RDV), a nucleoside analog originally developed for treating Ebola virus along with several other Food and Drug Administration (FDA)-approved drugs, is being evaluated in patients with COVID-19: including lopinavir (LPV) boosted by ritonavir, chloroquine (CLQ), favipiravir (FPV), and interferon (Beigel et al., 2020; Boulware et al., 2020; Cao et al., 2020; Dong et al., 2020; Touret and de Lamballerie, 2020). Reports on the clinical efficacies of these drugs are pending; however, it would be prudent to have a pipeline of additional drug candidates available for clinical trials.

In this study, we screened a panel of already approved drugs in a SARS-CoV-2 infection cell culture assay and identified two, cepharanthine (CEP) and nelfinavir (NFV), that showed more potent antiviral activity compared to RDV and other drugs currently being trialed. Our *in vitro*, *in silico*, and cell culture analyses demonstrate that CEP and NFV inhibit SARS-CoV-2 entry and RNA replication, respectively. Their different modes of action provided synergistic antiviral effects. We also mathematically predicted the potential antiviral efficacy of the single treatment of either CEP or NFV and its combination in clinical settings. These data cumulatively provide evidence for anti-SARS-CoV-2 potentials of CEP and NFV.

## Results

### Cepharanthine and nelfinavir inhibit SARS-CoV-2 infection

**Figure 1:**
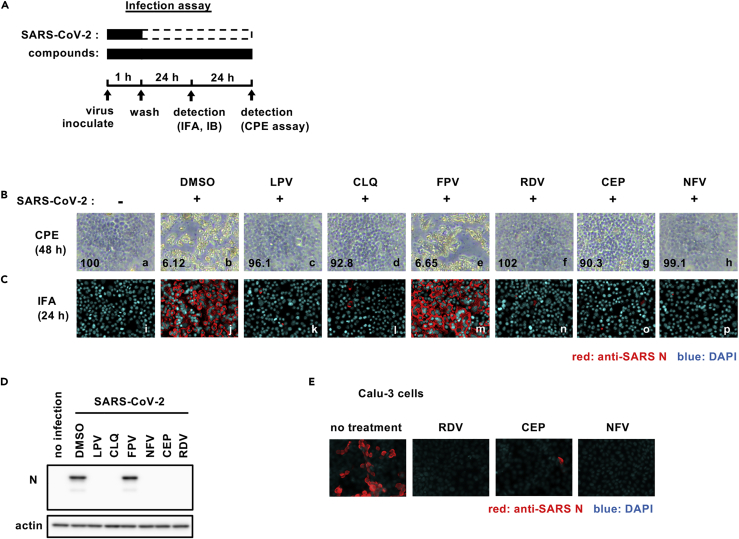
Cepharanthine (CEP) and nelfinavir (NFV) inhibit SARS-CoV-2 infection (A) Schematic of the SARS-CoV-2 infection assay. VeroE6/TMPRSS2 cells were inoculated with SARS-CoV-2 in the presence of compounds. After washing out unbound virus, the cells were incubated with compounds for 24-48 hr. Cells were harvested for immunofluorescence (IFA) or immunoblot analyses of viral N protein at 24 hr, and cytopathic effects (CPEs) were observed at 48 hr after infection. Solid and dashed boxes indicate the periods with and without treatment, respectively. (B–E) (B) Virus-induced CPE following drug treatment was recorded at 48 hr after infection. The quantified survival cell numbers (relative percentage to the control) are also shown at the bottom. Immunofluorescence (C) and immunoblot (D) detection of viral N protein expression in the infected cells at 24 hr after infection, and the red and blue signals show N and DAPI, respectively. (E) Immunofluorescent detection of viral N protein in human lung epithelial-derived cell line, Calu-3 cells. Dimethyl sulfoxide (DMSO), 0.4%; lopinavir (LPV), 16 μM; chloroquine (CLQ), 16 μM; favipiravir (FPV), 32 μM; remdesivir (RDV), 20 μM (C and D) and 10 μM (B and E); CEP, 8 μM; NFV, 4 μM (B-D) and 8 μM (E). These data were from three independent experiments.

We established a cell-based drug screening system to identify compounds that protect cells from SARS-CoV-2-induced cytopathology (Figure 1A): VeroE6/TMPRSS2 cells were treated with compounds for 1 hr during inoculation with a clinical isolate of SARS-CoV-2 (Matsuyama et al., 2020) at a multiplicity of infection (MOI) of 0.01 (or 0.001 for the indicated assay). Unbound virus was removed by washing, and the cells were treated with compounds for 48 hr to assess cell viability (Figure 1A) (methods). SARS-CoV-2 replication in VeroE6/TMPRSS2 induced a cytopathic effect and to validate our assay we show that two compounds, LPV and CLQ, that were reported to inhibit SARS-CoV-2 infection (Choy et al., 2020; Pizzorno et al., 2020; Wang et al., 2020a) reduced virus-induced cytopathicity (Figure 1B, compare b and c, d). After screening 306 FDA/European Medicines Agency/Pharmaceuticals and Medical Devices Agency-approved drugs, we identified compounds that protected cell viability by 20-fold compared with a dimethyl sulfoxide solvent control (methods). Among these, we selected to study CEP and NFV as candidates showing the greatest anti-cytopathic activity (Figures 1B–g, h). CEP is a Stephania-derived alkaloid extract with anti-inflammatory and anti-oxidative activities, and NFV targets human immunodeficiency virus protease (Bailly, 2019; Kao et al., 2015; Markowitz et al., 1998). To confirm and extend these observations, we assessed SARS-CoV-2-encoded N protein expression 24 hr after inoculation by immunofluorescence (Figure 1C, red) and immunoblotting (Figure 1D). Both CEP and NFV significantly reduced N protein expression along with the positive control drug candidates LPV, CLQ, and RDV. We confirmed that CEP and NFV inhibit SARS-CoV-2 proliferation in a human-derived lung epithelial cell line Calu-3 cells (Figure 1E).

### Dose-response curve for the anti-SARS-CoV-2 activity of cepharanthine and nelfinavir

**Figure 2:**
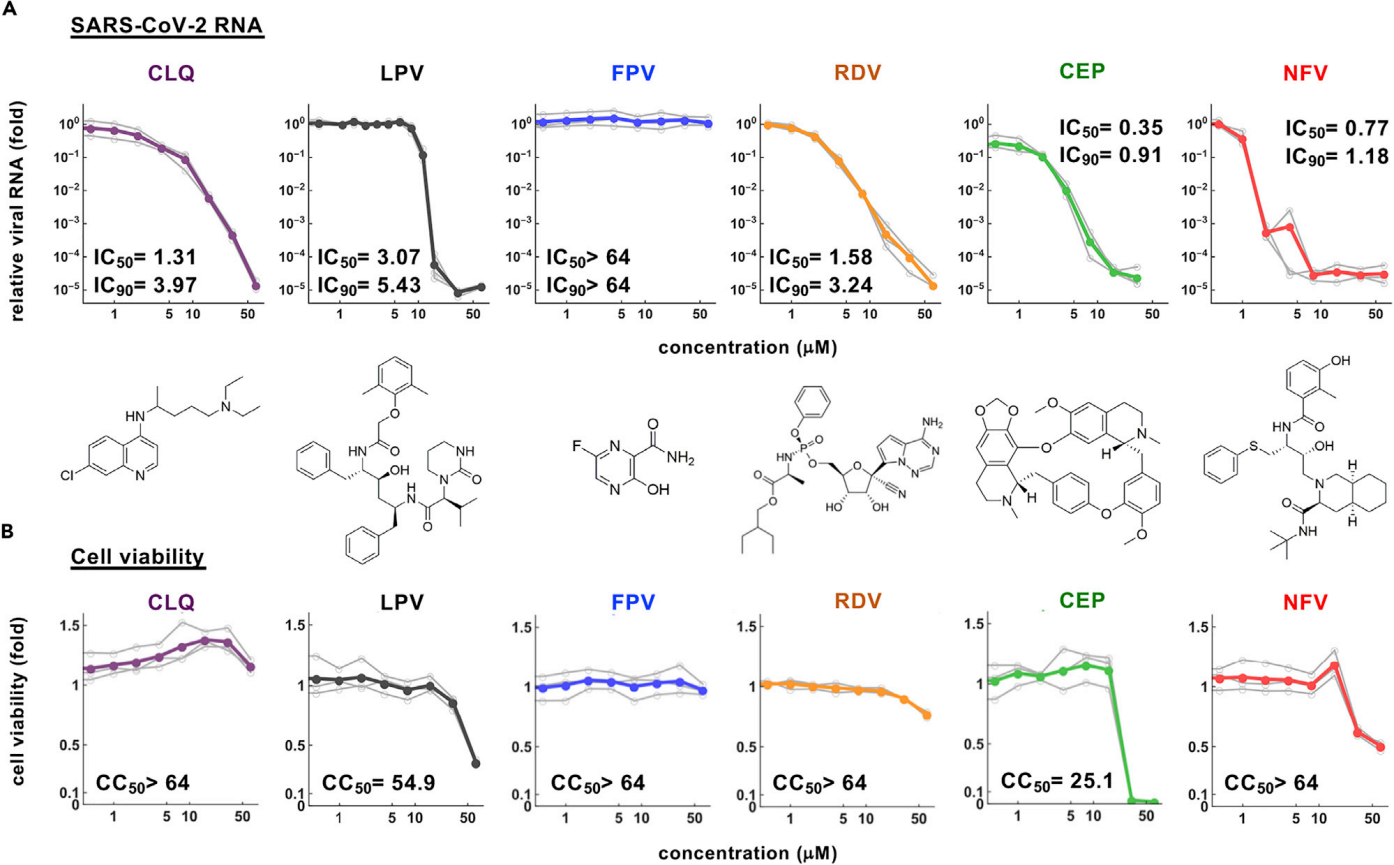
Dose-response curves for the antiviral activity of CEP and NFV (A and B) Dose-response curves for compounds. In (A), secreted viral RNA at 24 hr after inoculation was quantified and plotted against drug concentration and chemical structures shown below each graph (for CEP, the structure of the major component is shown). In (B), viability of cells treated with the compounds was quantified by MTT assay. IC_50_, IC_90_, and CC_50_ values were estimated by median effect model and are shown. These data were from three independent experiments.

To extend these observations, we quantified the effect of these compounds on secreted viral RNA and cell viability at 24 hr after infection. CEP and NFV significantly reduced viral RNA levels in a dose-dependent manner to 0.001–0.01% of the untreated control infections (Figure 2A). As expected, the positive control compounds (CLQ, LPV, and RDV) inhibited viral RNA, whereas FPV up to 64 μM showed negligible antiviral activity, consistent with previous reports (Choy et al., 2020; Jeon et al., 2020; Wang et al., 2020a). In parallel, we also assessed cell viability and noted cell death at high drug concentrations up to 64 μM (Figure 2B). The concentrations of drugs required to inhibit 50% (IC_50_) or 90% (IC_90_) of virus proliferation along with their 50% cytotoxicity (CC_50_) were estimated by median effect model and are listed in Figures 2A and 2B. These experiments highlight a >70-fold window (CC_50_/IC_50_) where CEP and NFV can inhibit SARS-CoV-2 proliferation with minimal toxicity. In summary, our screen identified two compounds that inhibit SARS-CoV-2 infection with high potency.

### Cepharanthine and nelfinavir have different modes of action

**Figure 3:**
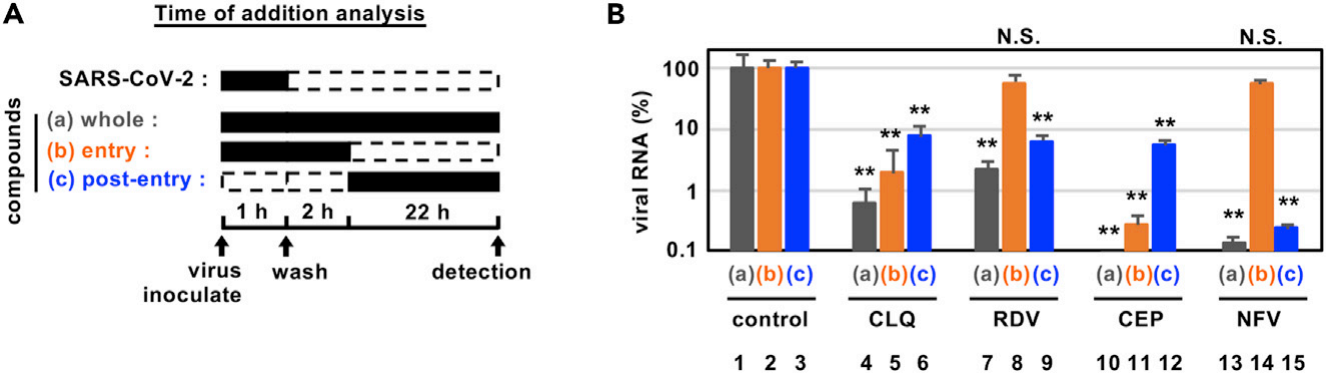
Antiviral mechanism of action for CEP and NFV (A and B) Time-of-addition analysis to examine steps in SARS-CoV-2 life cycle. (A) shows the schematic of the time-of-addition analysis. Compounds were added at different times (a, whole; b, entry; or c, post-entry): (A) presentation during the 1h virus inoculation step and maintained throughout the 24 hr infection period (whole life cycle); (B) present during the 1 hr virus inoculation step and for an additional 2 hr and then removed (entry); or (C) added after the inoculation step and present for the remaining 22 hr of infection (post-entry). Solid and dashed boxes indicate the periods with and without treatment, respectively. In (B), the antiviral activities of each compound under the various protocols are estimated by quantifying the levels of secreted viral RNA at 24 hr after inoculation (B; mean ± SD). RDV, 15 μM; CLQ, 15 μM; CEP, 8.2 μM; NFV, 4 μM. These data were from three independent experiments. ∗∗P < 0.01; N.S., not significant (Student's t-test)

To determine how these compounds impact on the viral replicative life cycle, we performed a time-of-addition assay (Figure 3A). We measured the antiviral activity of drugs added at different times: (a) present during the 1 hr virus inoculation step and maintained throughout the 24 hr infection period (“whole life cycle”); (b) present during the 1 hr virus inoculation step and for an additional 2 hr and then removed (“entry”); or (c) added after the inoculation step and present for the remaining 22 hr of infection (“post-entry”). CLQ, a known modulator of intracellular pH that inhibits virus entry (Akpovwa, 2016), was recently reported to inhibit SARS-CoV-2 (Liu et al., 2020a; Wang et al., 2020a), and we confirmed its activity in the early stages of infection (Figure 3B, lane 5). Since this assay allows multiple rounds of re-infection, entry inhibitors can show antiviral effects when added post-entry as in protocol (c) (Figure 3B, lane 6). RDV was previously reported to inhibit the process for intracellular viral replication (Wang et al., 2020a), and we confirmed this mode of action showing a reduction in viral RNA levels with a negligible effect on virus entry (Figure 3B, lane 8). This assay identified that CEP targeted the virus entry phase (Figure 3B, lanes 11) while NFV clearly inhibited the post-entry process (Figure 3B, lanes 15).

### Cepharanthine inhibits SARS-CoV-2 binding

**Figure 4:**
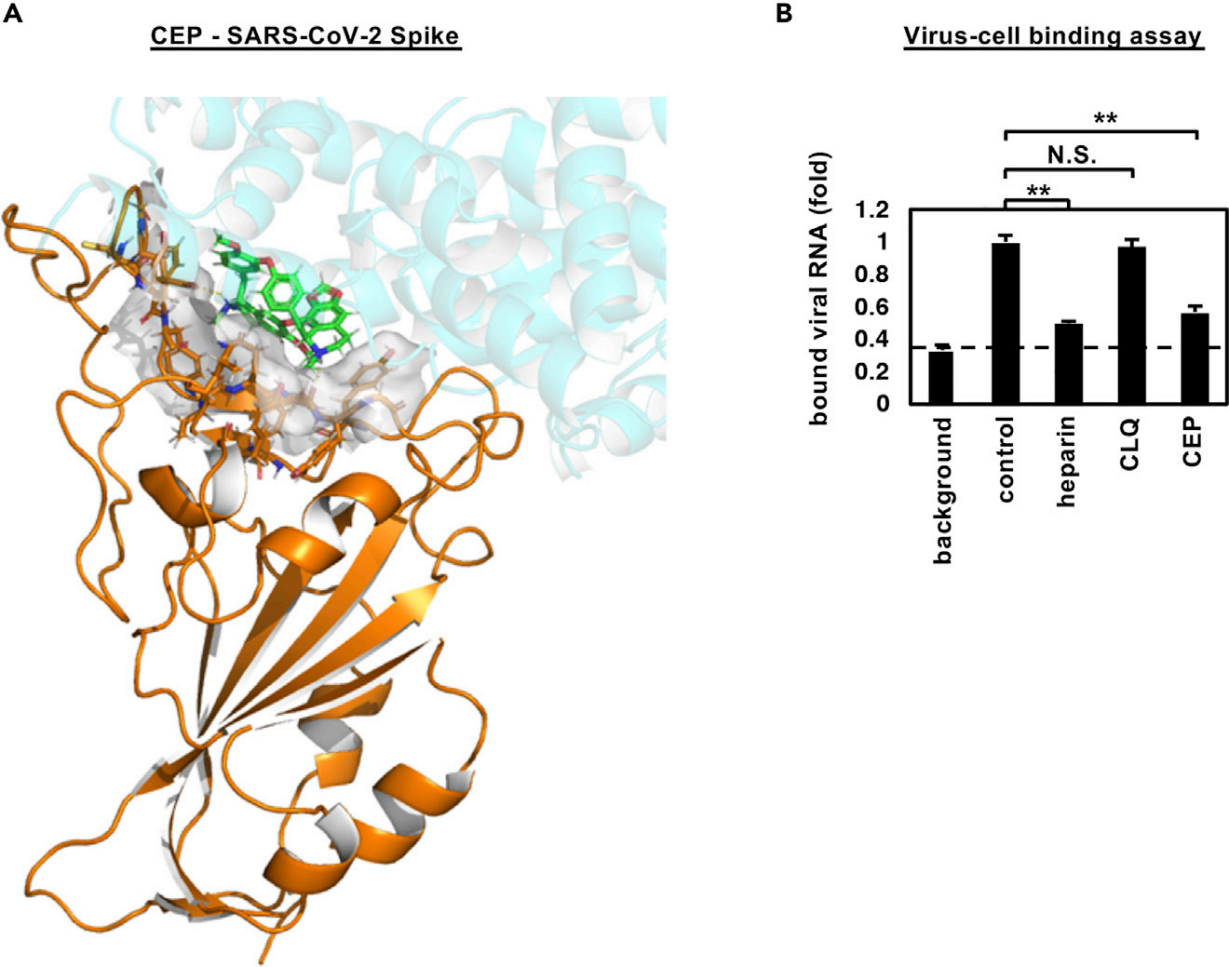
CEP inhibits SARS-CoV-2 cell binding (A) Predicted binding of CEP molecule to SARS-CoV-2 spike protein. Spike protein, CEP molecule, and protein binding site residues around CEP within 4 Å are shown in cartoon representation colored in orange, green stick, and surface representation, respectively. An overlapping view of the ACE2 with CEP is shown in semi-transparent cartoon representation colored in cyan. (B) Virus-cell binding assay. VeroE6/TMPRSS2 cells were incubated with virus (MOI = 0.001) in the presence of the indicated compounds for 30 min at 4°C to allow virus-cell binding. After extensive washing, cell-bound viral RNA was quantified, where the background depicts residual viral inocula in the absence of cells (B; mean ± SD). These data were from three independent experiments. ∗∗p < 0.01; N.S., not significant (Student's t-test)

*In silico* docking simulation shows that CEP molecules can bind the SARS-CoV-2 spike (S) protein and interfere with S engagement to its receptor, angiotensin-converting enzyme 2 (ACE2) (Lan et al., 2020; Walls et al., 2020; Wang et al., 2020b) (Figure 4A, green stick: CEP molecule, orange: S, semi-transparent cyan: ACE2). The docking model suggests that the NH of the piperidine ring of CEP molecules forms a hydrogen bond with the side chain carboxyl group of Glu484 and the backbone carbonyl group of Ser494, and the aromatic rings are in close contact with the aromatic residues (Tyr449, Tyr453, Tyr489, and Phe490) at the binding interface with ACE2. Binding free energy of CEP molecules was estimated as −24.26 kcal/mol using molecular mechanics generalized Born surface area calculation (Schrödinger, LLC). To assess this model, we investigated whether CEP inhibits SARS-CoV-2 particle binding to the cell surface or subsequent internalization into cells. We measured viral binding to cells by pre-chilling cells to prevent particle endocytosis and quantified cell-bound virus particles by quantitative PCR (qPCR) of viral RNA. CEP significantly inhibited SARS-CoV-2 binding to cells, whereas CLQ that targets intracellular trafficking pathways (Liu et al., 2020a) had a negligible effect (Figures 4B and S1). Viruses frequently exploit cellular heparan sulfate proteoglycans to initiate cell attachment, and heparin shows broad-spectrum inhibition of virus-cell attachment (De Clercq, 1998; Lang et al., 2011). As expected, heparin significantly blocked SARS-CoV-2 particle attachment to the cells (Figure 4B). These data demonstrate that CEP inhibits SARS-CoV-2 particle binding to cells.

### Nelfinavir potently targets SARS-CoV-2 main protease

**Figure 5:**
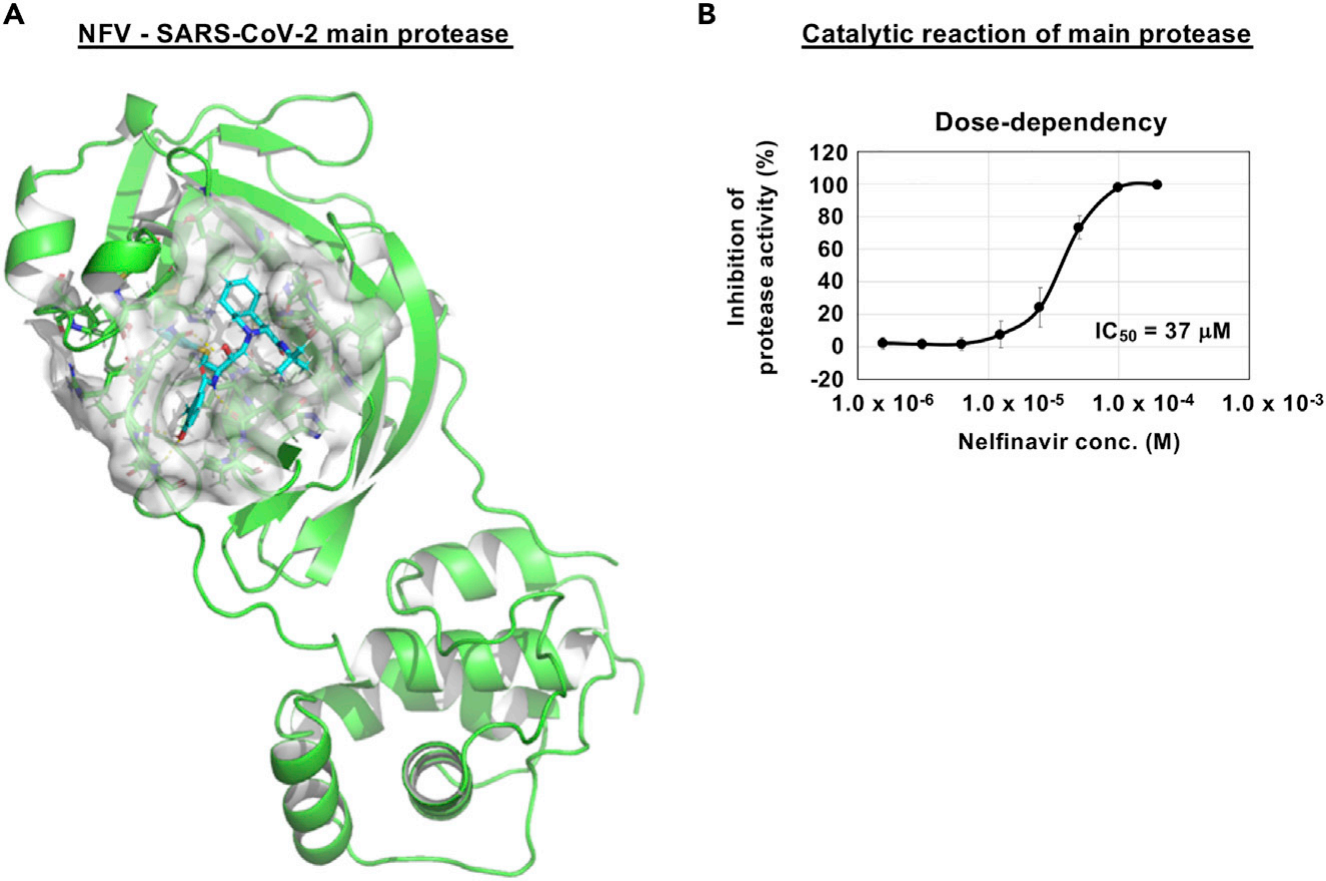
NFV potentially targets SARS-CoV-2 main protease (A) Predicted binding of NFV to SARS-CoV-2 main protease. Representation of SARS-CoV-2 main protease (green), NFV molecule (cyan stick), and protease binding site residues around NFV within 4 Å (surface representation) is shown. (B) Dose-dependent inhibition curves for NFV on the catalytic activity of the SARS-CoV-2 main protease. The IC_50_ is also shown. (B; mean ± SD)

We conducted *in silico* docking simulation screenings to identify compounds from an approved library that interact with the SARS-CoV-2-encoded main protease (methods). Interestingly, NFV was identified among the top 1.5% ranking compounds (Figure 5A, cyan stick: NFV, green: main protease). Our docking model predicts that NFV interacts with the SARS-CoV-2 protease active site pocket and would block substrate recruitment (Figure 5A). To assess this model, we evaluated the activity of recombinant SARS-CoV-2 main protease using an *in vitro* protease assay (methods). We showed that NFV inhibited the catalytic activity of the SARS-CoV-2-encoded main protease in a dose-dependent manner, and its IC_50_ was calculated to be 37 μM (Figure 5B). These *in vitro* and *in silico* data suggest that NFV potentially targets the main protease, but its inhibition activity is likely to be weaker than that to block SARS-CoV-2 replication.

### Synergy between cepharanthine and nelfinavir in blocking SARS-CoV-2 infection

**Figure 6:**
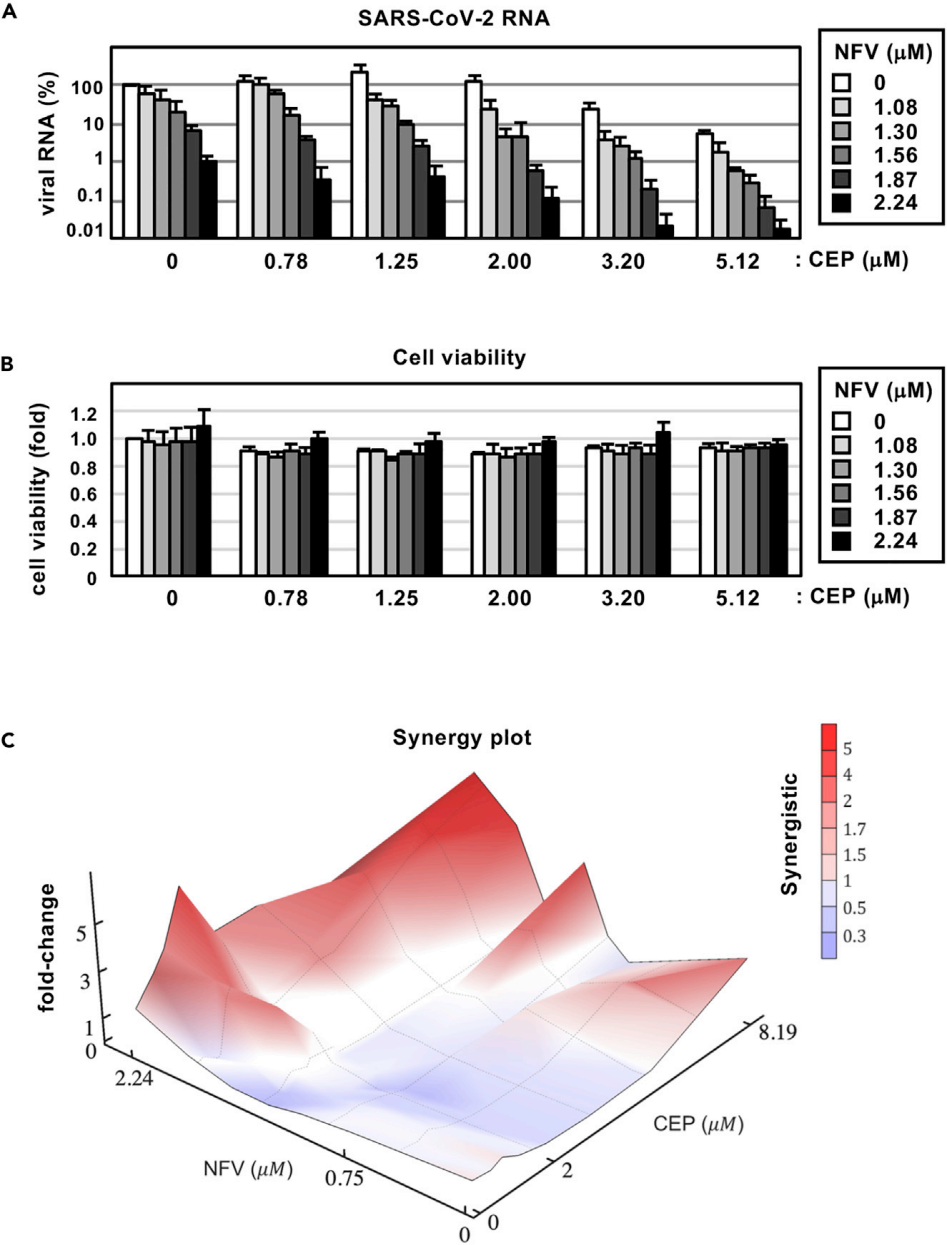
Combination treatment with CEP and NFV (A) Dose-response curve of CEP/NFV co-treatment in the infection experiment (MOI = 0.001). Extracellular viral RNA levels at 24 hr after infection were quantified and plotted against concentrations of CEP (0.78, 1.25, 2.00, 3.20, and 5.12 μM: 1.6-fold serial dilution) and NFV (1.08, 1.30, 1.56, 1.87, and 2.24 μM: 1.2-fold serial dilution). (B) Cell viability upon co-treatment with compounds. (C) The three-dimensional interaction landscapes of CEP and NFV were evaluated based on the Bliss independence model. Red and blue colors on the contour plot indicate synergy and antagonism, respectively. These data were from three independent experiments (A, B; mean ± SD).

Both CEP and NFV show anti-SARS-CoV-2 activity at the concentration ranges observed in patients, where the serum C_max_ of both drugs is 2.3 and 6.9 μM (by administration of 500 mg NFV orally and of 100 mg CEP by intravenous injection), respectively (Markowitz et al., 1998; Yasuda et al., 1989). Since CEP and NFV have different mode of actions, we examined their potential for synergistic effects. Antiviral activity and cell viability were determined by qPCR enumeration of viral RNA and MTT activity, respectively, following treatment with each compound alone or in combination (Figures 6A and 6B). For these experiments, we infected cells with lower amounts of SARS-CoV-2 (MOI = 0.001) and treated compounds at more frequent points of concentrations than those used in our earlier assay, for securing an accurate estimation. Single treatment with CEP (see white bars in Figure 6A) or NFV (see bars at CEP 0 μM) reduced viral RNA in a dose-dependent manner and co-treatment further reduced viral RNA levels (Figure 6A): e.g. CEP (3.20 μM) or NFV (2.24 μM) alone reduced viral RNA to 6.3% or 5.8% of untreated control, respectively, however, when combined they reduced viral RNA level to 0.068%. Higher doses of the CEP/NFV combination (4 μM each) reduced the viral RNA to undetectable levels. We compared the observed experimental antiviral activity (Figures 6A and S2A) with theoretical predictions calculated using a classical Bliss independence method that assumes drugs act independently (Note S1, Figure S2B) (Greco et al., 1995; Koizumi and Iwami, 2014). The difference between the observed values and theoretical predictions suggests that CEP and NFV exhibit a synergistic activity over a broad range of concentrations (Figure 6C red: synergistic effect).

### Mathematical modeling for the impact of cepharanthine and nelfinavir on SARS-CoV-2 dynamics in clinical settings

**Figure 7:**
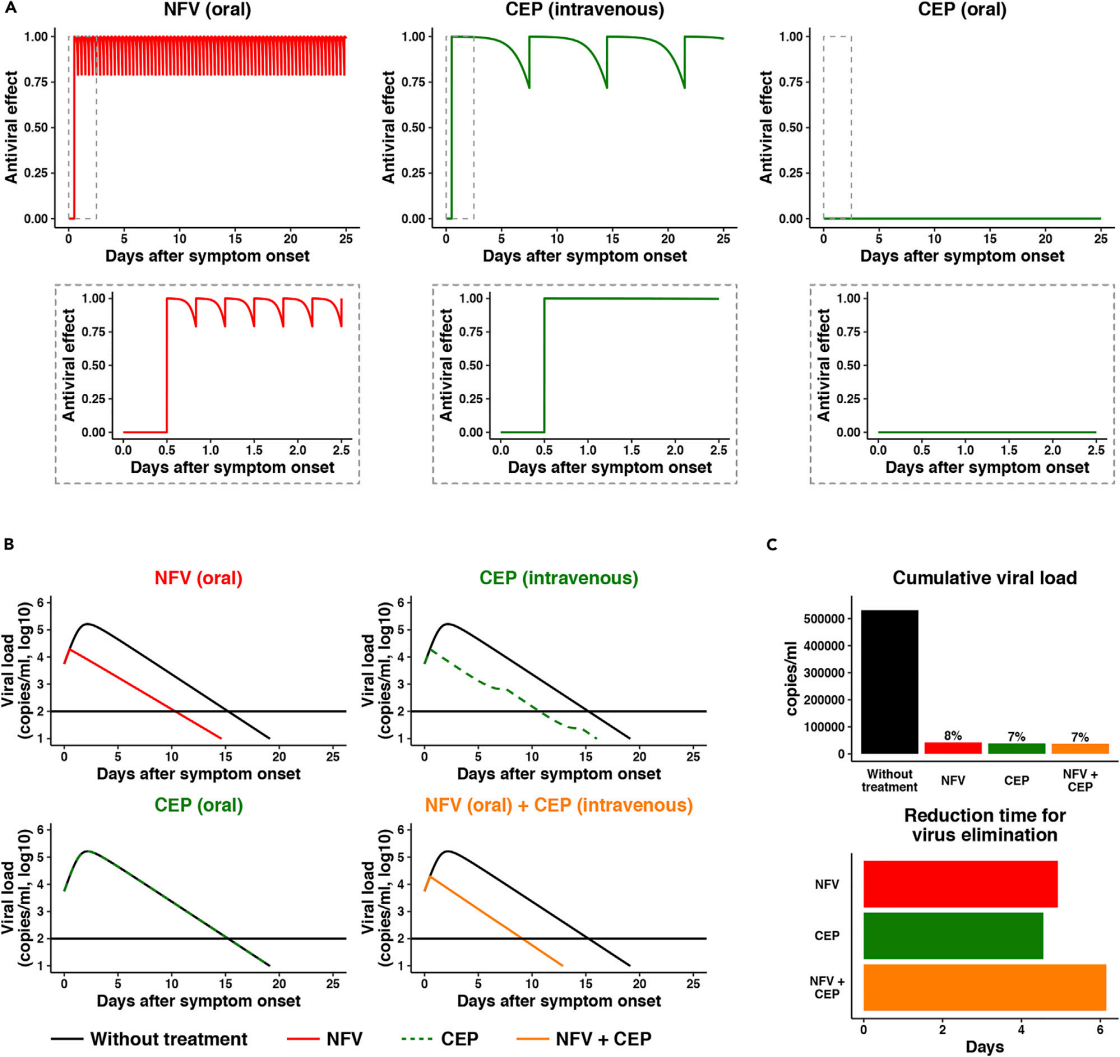
Mathematical prediction of the impact of CEP and NFV therapy on viral dynamics (A) The time-dependent antiviral effects of NFV (500 mg, TID, oral) and CEP [25 mg, intravenous drip or 10 mg, oral] predicted by pharmacokinetics/pharmacodynamics (PK/PD) model are shown, with enlarged views of the gray zones in upper panels. (B) Viral load dynamics in the presence or absence of NFV (oral), CEP (intravenous), CEP (oral), and NFV (oral)/CEP (intravenous) combined therapies predicted by pharmacokinetics/pharmacodynamics/viral dynamics (PK/PD/VD) models are shown. (C) The cumulative antiviral load [area under the curve in (B)] (upper) and the reduction time (days) for virus elimination (lower) with drug treatments [NFV (oral), CEP (intravenous), and the NFV (oral)/CEP (intravenous) combination] are shown.

Combining the published human clinical pharmacokinetics information for these drugs (Markowitz et al., 1998; Yasuda et al., 1989; Yokoshima et al., 1986) with our observed dose-dependent antiviral data, we can calculate the antiviral activity at the time after administration (Figure 7A: left, NFV oral; center, CEP intravenous drip; right: CEP oral). Here, we used the reported pharmacokinetic information for drug distribution in the lung as well as the time-dependent drug concentration in plasma and assumed that antiviral activity depends on drug concentration in the lung (Ford et al., 2004; Shetty et al., 1996; Twigg et al., 2010) (see supplemental information in detail). Based on the time-dependent antiviral activity of drug, we can model the impact on viral burden following drug administration (Figure 7B, Note S1, Figure S3). From the viral dynamics data in Figure 7B, we calculated the cumulative viral RNA burden (i.e., area under the curve of viral load) (Figure 7C, upper) and the time required to reduce the viral load to undetectable levels (Figure 7C, lower). Our modeling predicts that NFV monotherapy would reduce the cumulative viral load by 92.1% (Figure 7C, upper, red) and would require 10.3 days to eliminate virus (Figure 7B, upper left, red), 4.9 days shorter than untreated controls (Figure 7C, lower, red). In contrast, orally administered CEP shows a minimal effect on the viral load (Figure 7B, lower left, green), most likely reflecting low drug concentrations, while intravenous delivery of CEP reduces the cumulative viral load (Figures 7B and 7C, green) and shortens the period for virus elimination (Figure 7C, lower, green) because of achieving enough drug concentration (see Discussion). Importantly, co-administering NFV (oral) and CEP (intravenous drip) resulted in a more rapid decline in viral RNA, with undetectable levels 6.15 days earlier than untreated controls and 1.23 days earlier than NFV alone (Figure 7C, orange). Another advantage of combination treatment is discussed in discussion. In summary, our prediction shows the potential antiviral efficacy of NFV and CEP and its combined treatment that facilitates SARS-CoV-2 elimination.

## Discussion

Screening a panel of approved drugs identified two agents, CEP and NFV, that inhibit SARS-CoV-2 infection with the highest potencies in our screening. A recent study reported that CEP showed anti-SARS-CoV-2 activity, and the authors speculated that CEP targeted both entry and viral replication phase of the virus life cycle (Fan et al., 2020; Jeon et al., 2020). However, our time-of-addition studies along with viral binding and docking simulation analysis suggest that CEP predominantly inhibits virus-cell binding. We also have preliminary data by surface plasmon resonance analysis showing a potential interaction between CEP and the S protein, speculating its mode of action and which needs to be further analyzed in the future. These data are consistent with a previous paper reporting that CEP reduced the entry of another human coronavirus OC43 (Kim et al., 2019). There is a significant global effort to generate a COVID-19 vaccine that will target the SARS-CoV-2 S glycoprotein (Thanh Le et al., 2020). It is worthy of future investigation to examine whether CEP is effective to augment the antiviral activity of neutralizing antibodies. After the emergence of COVID-19 pandemic, *in silico* studies have been widely conducted to seek for anti-COVID-19 drugs and NFV was predicted for a potential to associate with SARS-CoV-2 life cycle (Huynh et al., 2020; Mittal et al., 2020; Mothay and Ramesh, 2020; Musarrat et al., 2020; Reiner et al., 2020). NFV was reported to inhibit the replication of another coronavirus, SARS-CoV (Liu et al., 2005; Wu et al., 2004; Yamamoto et al., 2004). Our study is consistent with the recent report showing the anti-SARS-CoV-2 activity of NFV, which has been published during the review process of this paper, although its mode of action and the prediction of antiviral effect in clinical settings were not analyzed (Ianevski et al., 2020). In addition to the identification of anti-SARS-CoV-2 activity of NFV from a chemical screening, our study showed that NFV inhibited SARS-CoV-2 replication with under μM order and inhibited the catalytic activity of main protease with lower activity, predicted by *in silico* modeling. Our data suggest that NFV potently inhibits the main protease and also possibly targets another factor. A non-infectious cell fusion system also reported that NFV inhibited SARS-CoV-2 spike-mediated membrane fusion at the concentration of over 10 μM (Musarrat et al., 2020), providing another possible antiviral activity of NFV. In addition, the observation that CEP and NFV target different steps in the viral life cycle supports the development of multidrug combination therapies for treating COVID-19.

Our mathematical modeling studies assess how drug candidates can suppress and eliminate SARS-CoV-2. Based on the reported lung distribution/concentration of CEP and NFV in patients, we predicted that NFV at clinical doses can maintain significant antiviral effects throughout the treatment period and reduce SARS-CoV-2 RNA burden that results in shortening the time required to eliminate infection. In contrast, we predict that oral administration of CEP will have limited antiviral effect due to its low concentration *in vivo*. However, intravenous delivery of CEP achieves higher drug concentrations especially accumulated in the lung (Yokoshima et al., 1986) that enables sustained antiviral activity. It is noteworthy that combining CEP with NFV further reduced the cumulative viral load and facilitated virus elimination. As the cumulative viral load in patients is likely to associate with disease progression and risk of new transmission (Liu et al., 2020b), such multidrug treatments will be of benefit to improve clinical outcome and to control the epidemic. In addition to potentiating antiviral effects, combination treatment can limit the emergence of viral drug resistance which is frequently reported for RNA viruses such as coronavirus. Limitations of this mathematical prediction are shown in limitations of the study section; however, our analysis warrants the further clinical trial for oral NFV treatment in Japan (jRCT2071200023).

Several *in vivo* SARS-CoV-2 infection systems were recently reported: nonhuman primates, ferrets, hamsters, transgenic mice overexpressing human ACE2, and wild-type mice infected with mouse-adapted virus (Bao et al., 2020; Gao et al., 2020; Kim et al., 2020; Munster et al., 2020; Rockx et al., 2020). Given the urgency of the COVID-19 pandemic, we believe that a lack of *in vivo* validation should not preclude the clinical assessment of new antiviral agents. We here propose CEP and NFV as potential antiviral drug candidates against COVID-19, and thus, NFV is under clinical evaluation in a multicenter randomized controlled trial in Japan (jRCT2071200023).

### Limitations of the study

In this study, we mainly used VeroE6/TMPRSS2 cells and applied the dose-dependent antiviral activity in these cells (Figure 2A) to predict the drug efficacy in patients (Figure 7). More physiologically relevant cell models such as primary human respiratory/lung cells in air-liquid interface culture and organoids or presumably *in vivo* infection models would be needed to strengthen the data. As well, our mathematical prediction was based on the total drug concentration in the lung, although free drug that does not non-specifically bind to proteins is believed to be pharmacologically active. There is no information available on the free CEP and NFV concentration in the lung tissue.

### Resource availability

#### Lead contact

Further information and requests for resources and reagents should be directed to and will be fulfilled by the lead contact, Koichi Watashi: kwatashi@nih.go.jp.

#### Materials availability

This study did not generate new unique materials.

#### Data and code availability

All data are included in the article and supplemental information and any additional information will be available from the lead contact upon request.

## Methods

All methods can be found in the accompanying transparent methods supplemental file.

## Supplemental information

Document S1. Transparent methods, Figures S1–S3, Tables S1–S3, and Note S1

## Footnotes

Supplemental information can be found online at https://doi.org/10.1016/j.isci.2021.102367.
