# Supplementary material for: Potential anti-COVID-19 agents, cepharanthine and nelfinavir, and their usage for combination treatment
Source: iScience. 2021 Mar 26;24(4):102367. doi: 10.1016/j.isci.2021.102367 (PMC7997640; doi:10.1016/j.isci.2021.102367)
Supplement: Document S1. Transparent methods, Figures S1–S3, Tables S1–S3, and Note S1 [file mmc1.pdf]

iScience, Volume *24*

Supplemental information

Potential anti-COVID-19 agents, cepharanthine and nelfinavir, and their usage for combination treatment

Hirofumi Ohashi, Koichi Watashi, Wakana Saso, Kaho Shionoya, Shoya Iwanami, Takatsugu Hirokawa, Tsuyoshi Shirai, Shigehiko Kanaya, Yusuke Ito, Kwang Su Kim, Takao Nomura, Tateki Suzuki, Kazane Nishioka, Shuji Ando, Keisuke Ejima, Yoshiki Koizumi, Tomohiro Tanaka, Shin Aoki, Kouji Kuramochi, Tadaki Suzuki, Takao Hashiguchi, Katsumi Maenaka, Tetsuro Matano, Masamichi Muramatsu, Masayuki Saijo, Kazuyuki Aihara, Shingo Iwami, Makoto Takeda, Jane A. McKeating, and Takaji Wakita

Supplemental Information

Potential anti-COVID-19 agents, Cepharanthine and Nelfinavir, and their usage for combination treatment

Hirofumi Ohashi[1,2,¶], Koichi Watashi[1,2,3,4,29,¶*], Wakana Saso[1,5,6,¶], Kaho Shionoya[1,2], Shoya Iwanami[7], Takatsugu Hirokawa[8,9,10], Tsuyoshi Shirai[11], Shigehiko Kanaya[12], Yusuke Ito[7], Kwang Su Kim[7], Takao Nomura[13], Tateki Suzuki[14], Kazane Nishioka[1,2], Shuji Ando[15], Keisuke Ejima[16], Yoshiki Koizumi[17], Tomohiro Tanaka[18], Shin Aoki[18,19], Kouji Kuramochi[2], Tadaki Suzuki[20], Takao Hashiguchi[14], Katsumi Maenaka[13,21,22], Tetsuro Matano[5,6], Masamichi Muramatsu[1], Masayuki Saijo[15], Kazuyuki Aihara[23], Shingo Iwami[4,7,24,25,26], Makoto Takeda[27], Jane A. McKeating[28], Takaji Wakita[1]

[1]Department of Virology II, National Institute of Infectious Diseases, Tokyo 162-8640, Japan,

[2]Department of Applied Biological Science, Tokyo University of Science, Noda 278-8510, Japan,

[3]Institute for Frontier Life and Medical Sciences, Kyoto University, Kyoto 606-8507, Japan,

[4]MIRAI, JST, Saitama 332-0012, Japan,

[5]The Institute of Medical Science, The University of Tokyo, Tokyo 108-8639, Japan,

[6]AIDS Research Center, National Institute of Infectious Diseases, Tokyo 162-8640, Japan,

[7]Department of Biology, Faculty of Sciences, Kyushu University, Fukuoka 812-8581, Japan,

[8]Cellular and Molecular Biotechnology Research Institute, National Institute of Advanced Industrial Science and Technology, Tokyo 135-0064, Japan,

[9]Division of Biomedical Science, Faculty of Medicine, University of Tsukuba, Tsukuba 305-8575, Japan,

[10]Transborder Medical Research Center, University of Tsukuba, Tsukuba 305-8575, Japan,

[11]Faculty of Bioscience, Nagahama Institute of Bio-Science and Technology, Nagahama 526-0829, Japan,

[12]Graduate School of Science and Technology, Nara Institute of Science and Technology, Ikoma 630-0192, Japan,

[13]Center for Research and Education on Drug Discovery, Faculty of Pharmaceutical Sciences, Hokkaido University, Sapporo 060-0812, Japan,

[14]Department of Virology, Faculty of Medicine, Kyushu University, Fukuoka 812-8582, Japan,

[15]Department of Virology I, National Institute of Infectious Diseases, Tokyo 162-8640, Japan,

[16]Department of Epidemiology and Biostatistics, Indiana University School of Public Health-Bloomington, IN 47405, USA,

[17]National Center for Global Health and Medicine, Tokyo162-8655, Japan,

[18]Faculty of Pharmaceutical Sciences, Tokyo University of Science, Noda 278-8510, Japan,

[19]Research Institute for Science and Technology, Tokyo University of Science, Noda 278-8510, Japan,

[20]Department of Pathology, National Institute of Infectious Diseases, Tokyo 162-8640, Japan,

[21]Laboratory of Biomolecular Science, Faculty of Pharmaceutical Sciences, Hokkaido University, Sapporo 060-0812, Japan,

[22]Global Station for Biosurfaces and Drug Discovery, Center for Life Innovation, Hokkaido University, Sapporo 060-0812, Japan,
[23]International Research Center for Neurointelligence, The University of Tokyo Institutes for Advanced Study, The University of Tokyo, Tokyo 113-8654, Japan,
[24]Institute for the Advanced Study of Human Biology (ASHBi), Kyoto University, Kyoto 606-8501, Japan,
[25]NEXT-Ganken Program, Japanese Foundation for Cancer Research (JFCR), Tokyo 135-8550, Japan,
[26]Science Groove Inc., Fukuoka 810-0041, Japan,
[27]Department of Virology III, National Institute of Infectious Diseases, Tokyo 208-0011, Japan,
[28]Nuffield Department of Medicine, University of Oxford, Oxford OX3 7FZ, UK.
[29]Research Center for Drug and Vaccine Development, National Institute of Infectious Diseases, Tokyo 162-8640, Japan

¶These authors contributed equally to this work

Transparent Methods
Supplemental Note
Supplemental Figure S1-S3
Supplemental Table S1-S3
Supplemental References

Transparent Methods

Cell culture. VeroE6/TMPRSS2 cells [VeroE6 cells overexpressing transmembrane protease, serine 2 (TMPRSS2) (Matsuyama et al., 2020) were cultured in Dulbecco's modified Eagle's medium (DMEM; Life Technologies) supplemented with 10% fetal bovine serum (FBS; Cell Culture Bioscience), 100 units/mL penicillin, 100 μg/mL streptomycin, 10 mM HEPES (pH 7.4), and 1 mg/mL G418 (Nacalai) at 37˚C in 5% CO_2. During the infection assay, 10% FBS was replaced with 2% FBS and G418 removed. Calu-3 cells were cultured in above medium supplemented with 10% FBS, 100 units/mL penicillin, and 100 μg/mL streptomycin.

Reagents. All the reagents were purchased from Selleck, Enzo Life Sciences, Cayman Chemical, Sigma, MedChemExpress, TCI or kindly donated by pharmaceutical companies (Abbvie, Alps Pharmaceutical, Asahi Kasei Pharma, Astellas Pharma, Bayer, Boehringer Ingelheim, Bristol-Myers Squibb, Chugai Pharmaceutical, Daiichi Sankyo, EA Pharma, Fujifilm Toyama Chemical, Japan Tobacco, Kakenshoyaku, Kissei Pharmaceutical, Kowa, Kyorin Pharmaceutical, Kyowa Pharmaceutical Industry, Maruho, Mitsubishi Tanabe Pharma, Mochida Pharmaceutical, Novartis, Sanofi, SBI Pharmaceuticals, Shionogi, Sumitomo Dainippon Pharma, Sun Pharma, Takeda Pharmaceutical, Teva Takeda Pharma). Note that throughout in this study we used the pharmaceutical preparation of Cepharanthine (kindly provided by Medisa Shinyaku Inc, a subsidiary of Sawai Pharmaceutical), which is a Stephania-derived alkaloid extract containing 19.5-33.5% Cepharanthine molecule as the major component.

Infection assay. SARS-CoV-2 was handled in a biosafety level 3 (BSL3). We used the SARS-CoV-2 Wk-521 strain, a clinical strain isolated from a COVID-19 patient, and obtained viral stocks by infecting VeroE6/TMPRSS2 cells (Matsuyama et al., 2020). Virus infectious titers were measured by inoculating cells with a 10-fold serial dilution of virus and cytopathology measured to calculate $TCID_{50}$/ml (Matsuyama et al., 2020). For the infection assay, VeroE6/TMPRSS2 cells were inoculated with virus at an MOI of 0.01 (Fig. 1, 2, and 3B, except for 0.001 in Fig. 1B) for 1 h and unbound virus removed by washing. In Fig. 4B, 6, and S1, we used 0.001 of MOI to avoid possible saturation of virus binding/replication. Cells were cultured for 24 h prior to measuring extracellular viral RNA or detecting viral encoded N protein, and cytopathic effects (CPE) after 48 h. Compounds were added during virus inoculation (1 h) and replenished after washing (24 or 48 h) except for time of addition assay. Infection assay with Calu-3 cells was performed by incubation with virus at an MOI of 0.04 for 3 h. N protein was detected at 72 h post-inoculation.

For the time of addition assay, we added compounds with three different timings (Fig. 3A): (a) present during the 1 h virus inoculation step and maintained throughout the 24 h infection period (**whole life cycle**); (b) present during the 1 h virus inoculation step and for an additional 2 h and then removed (**entry**); or (c) added after the inoculation step and present for the remaining 22 h of infection (**post-entry**). Inhibitors of viral replication are expected to show antiviral activity in (a) and (c), but not (b), while entry inhibitors (e.g. chloroquine) reduce viral RNA in all three conditions (In c, addition of entry

inhibitors after inoculation inhibits re-infection and thus decreases viral RNA) (Wang et al., 2020).

Quantification of viral RNA. We mainly quantified viral RNA to measure the antiviral activity of drugs. Viral RNA was extracted with a QIAamp Viral RNA mini, RNeasy mini kit (QIAGEN), or MagMax Viral/Pathogen II Nucleic Acid Isolation kit (Thermo Fisher Scientific) and quantified by real time RT-PCR analysis with a one-step qRT-PCR kit (THUNDERBIRD Probe One-step qRT-PCR kit, TOYOBO) using 5'- ACAGGTACGTTAATAGTTAATAGCGT-3', 5'- ATATTGCAGCAGTACGCACACA-3', and 5'-FAM-ACACTAGCCATCCTTACTGCGCTTCG-TAMRA-3' (E-set) (Corman et al., 2020) or 5'-AAATTTTGGGGAVVAGGAAC-3', 5'-TGGCAGCTGTGTAGGTCAAC-3', and 5'-FAM-ATGTCGCGCATTGGCATGGA-TMARA-3' (N2-set). Detection limit of SARS-CoV-2 RNA for N2-set was 38.1 cycle as C_t cycle.

Detection of viral N protein. Viral protein expression was detected using a rabbit anti-SARS-CoV N antibody (Mizutani et al., 2004) with AlexaFluor 568 anti-rabbit IgG or anti-rabbit IgG-HRP (Thermo Fisher) by indirect immunofluorescence or immunoblot analyses as previously reported (Ohashi et al., 2018).

Cell viability and virus induced cytopathology. Cell viability (Fig. 2B) was determined by MTT assay as previously reported (Ohashi et al., 2018). Virus-induced cytopathology was observed by microscopy at 48 h post-infection as previously reported (Matsuyama et al., 2020). Quantification of cell number (Fig. 1B) was performed with a high-content imaging system as shown below.

Chemical screening. We screened an FDA/EMA/PMDA-approved chemical library composed of 306 compounds by the following cytopathic effect assay to augment the throughput. Cells were treated with compounds at 8, 16, or 30 μM for 1 h during virus inoculation and for up to 72 h post-inoculation. The cells were then fixed with 4% paraformaldehyde and stained with DAPI to count viable cells using a high-content imaging system (ImageXpress Micro Confocal, Molecular Devices). Compounds that protected cells from virus-induced cytopathology and showed cell survival more than 20-fold of the control were selected as hits. Among 306 tested compounds, Cepharanthine, Lopinavir, Loteprednol, Nelfinavir, and Rapamycin were identified as hits. Lopinavir is currently being evaluated in clinical trials for treatment of COVID-19 (Cao et al., 2020). As Loteprednol and Rapamycin are steroid and immunosuppressant, respectively, which suppress immune response, we focused on Cepharanthine and Nelfinavir in this study.

Virus-cell binding assay. SARS-CoV-2 was preincubated with the indicated compounds at 37˚C for 30 min. The SARS-CoV-2 (MOI=0.001) was exposed to VeroE6/TMPRSS2 cells at 4˚C for varying time (5, 15, 30 and 50 min). After extensive wash, total RNA was extracted from cells and viral RNA was quantified by real time RT-PCR to measure cell-bound virus. In this assay, signal/noise ratio of detected RNA for the control with 30 min incubation (Fig. 4B) was more than 300-fold.

Docking simulation of compound binding with a target protein. The crystal structure of the main protease and spike protein were obtained from Protein Data Bank (6LU7 (Jin et al., 2020) and 6M0J (Lan et al., 2020)) and refined for docking simulations using the Protein Preparation Wizard Script within Maestro (Schrödinger, LLC). We carried out *in silico* library screening based on the active site pocket of the main protease using combined molecular docking with a protein-ligand interaction fingerprint scoring method against 8,085 known drugs obtained from the KEGG-Drug database (Kanehisa and Goto, 2000). For all compounds ionization and energy minimization were performed by the OPLS3 force field in the LigPrep Script of Maestro (Schrödinger, LLC). These minimized structures were used as input structures for docking simulations. Docking simulations were performed using the Glide (Friesner et al., 2004; Halgren et al., 2004) SP docking program (Schrödinger, LLC) with a grid box defined by N3 inhibitor molecule for main protease and ACE2 binding interface residues for spike protein using BioLuminate (Schrödinger, LLC)

In vitro SARS-CoV-2 protease assay. The SARS-CoV-2 encoded main protease was purchased from BPS Bioscience, Inc. (USA). The synthesized fluorogenic peptide Ac-Abu-Tle-Leu-Gln-MCA (Rut et al., 2020) was kindly provided by Peptide Institute, Inc. (Japan), and was used as a substrate for the proteolytic assay using the SARS-CoV-2 main protease. The assay was performed in the buffer (20 mM Tri-HCl, pH7.3, 100 mM NaCl, 1 mM DTT, 1 mM EDTA) containing 20 μM of the fluorogenic peptide and 200 nM of the protease. The protease and NFV were pre-incubated at 37°C for 30 min. The reaction was initiated by addition of the substrate and incubated for 30 min at 37°C. The fluorescence of Aminomethylcoumarin due to cleavage of the fluorogenic peptide was monitored at 460 nm with excitation at 380 nm on a fluorescence plate reader (En Spire, Perkin Elmer).

Mathematical analysis. Determination of synergism between NFV and CEP and simulation of virus dynamics as well as the calculation of IIP are shown in detail in Supporting Note.

Statistics. Statistical significance estimated using the two-tailed Student's t test (*$p<0.05$; **$p<0.01$; N.S., not significant).

Supplemental Note, related to Figure 2, 6, and 7.

Quantifying instantaneous inhibitory potential (IIP) from the dose-response curves of the drugs

The typical dose-response curves of a single antiviral drug can be analyzed using the following Hill function (Koizumi et al., 2017) (**Fig. 2A**):

$$f_u = \frac{1}{1 + \left(\frac{D}{IC_{50}}\right)^m}. \qquad (1)$$

Here, f_u represents the fraction of infection events unaffected by the drug (i.e., $1 - f_u$ equals the fraction of drug-affected events). D is the drug concentration, IC_{50} is the drug concentration that achieves 50% inhibition of activity, and m is the slope of the dose-response curve (i.e., Hill coefficient) (Koizumi et al., 2017). Dose-response curves for drugs with higher m values show stronger antiviral activity at the same normalized drug concentration so long as the drug concentration is higher than IC_{50} (**Fig. 2A**). Least-square regression approach was used to fit Eq.(1) to dose-response data and estimate the values of IC_{50} and m. Those estimated values for each drug against SARS-CoV-2 are summarized in **Table S1**.

Expected anti-SARS-CoV-2 effect of double-drug combinations by Bliss independence

We evaluated the effect of double-drug combinations for Bliss independence which is widely used to analyze drug combination data (Bliss, 1939; Kobayashi et al., 2014; Koizumi and Iwami, 2014; Tallarida, 2001). Bliss independence model assumes that each drug acts on different targets/mechanisms, and is defined as:

$$f_u^{\mathrm{Bcom}} = f_u^A(D) \times f_u^B(D), \qquad (3)$$

where f_u^{Bcom}, f_u^A and f_u^B are the fractions of infection events unaffected by the combined drugs A (i.e., Nelfinavir: NFV) and B (i.e., Cepharanthine: CEP) expected by the Bliss model, single drug A and single drug B defined by Eq. (1), respectively. Using Eq. (2), we expected the anti-SARS-CoV2 effects of combined drugs A and B, $1 - f_u^{\mathrm{Bcom}}$, from the anti-SARS-CoV-2 effects of the single drugs (**Fig. S2**).

However, the Bliss model ignores interactions in which drugs enhance each other effects. To address this point, we introduced the recent proposed model (Zimmer et al., 2016), called "dose model" considering the drug interactions, and further evaluated the expected antiviral effects (**Fig. S2**). This drug interaction is described by introducing interaction terms between drug pairs, that is, the "effective" concentration of drug A (i.e., NFV) and B (i.e., CEP), D_A^{com} and D_B^{com}, are defined as follows;

$$D_A^{\mathrm{com}} = D_A \left(1 + a_{AB} \frac{D_B^{\mathrm{com}}}{IC_{50}^B + D_B^{\mathrm{com}}}\right)^{-1}, \qquad D_B^{\mathrm{com}} = D_B \left(1 + a_{BA} \frac{D_A^{\mathrm{com}}}{IC_{50}^A + D_A^{\mathrm{com}}}\right)^{-1},$$

where D_A and D_B are the "true" concentrations, IC_{50}^A and IC_{50}^B are the concentrations that achieve 50% inhibition of activity, a_{AB} and a_{BA} are the interaction parameters for drug A and B, respectively. Note that IC_{50}^A and IC_{50}^B are corresponding to the estimations from the dose-response curves of a

single antiviral drug in combination treatment experiment, which is summarized in **Table S1**, and $a_{AB} = -0.462$ and $a_{BA} = 0.307$ are estimated from the dose-response curves of the double-drug combination. The dose model extended the Bliss model, thus, the expected anti-SARS-CoV-2 effect with effective concentration of drugs A and B (rather than the true concentrations) are calculated as $1 - f_u^{\mathrm{Dcom}}(D)$ and

$$f_u^{\mathrm{Dcom}} = f_u^A(D_A^{\mathrm{com}}) \times f_u^B(D_B^{\mathrm{com}}). \quad (4)$$

The dose model assumed that the effects of drugs on each other's effective doses are multiplicative.

PK/PD/VD model for single- and double-drug combinations against SARS-CoV-2 infection

Based on a standard viral dynamics (VD) model (Ikeda et al., 2016), to describe COVID-19 dissemination among susceptible target cells, we used the following simple mathematical model proposed in (Kim et al., 2020):

$$\frac{df(t)}{dt} = -\beta f(t)V(t), \quad (5)$$

$$\frac{dV(t)}{dt} = \gamma f(t)V(t) - \delta V(t), \quad (6)$$

where $f(t)$ and $V(t)$ are the ratio of uninfected target cells and the amount of virus, respectively. The parameters β, γ, and δ represent the rate constant for virus infection, the maximum rate constant for viral replication and the death rate of infected cells, respectively. All viral load data including Singapore and Zhuhai patients (Young et al., 2020; Zou et al., 2020) were simultaneously fitted using a nonlinear mixed-effect modelling approach, which uses the whole samples to estimate population parameters while accounting for inter-individual variation. The estimated parameters and initial values used here are summarized in **Table S3**.

To investigate the expected outcome for anti-SARS-CoV-2 therapies with single-drug, we conducted *in silico* experiments with the following PK/PD/VD model for replication inhibitor such as Nelfinavir (**Fig. 7**);

$$\frac{df(t)}{dt} = -\beta f(t)V(t), \quad (7)$$

$$\frac{dV(t)}{dt} = \big(1 - \varepsilon(t) \times H(t)\big)\gamma f(t)V(t) - \delta V(t), \quad (8)$$

and for entry inhibitor such as Cepharanthine;

$$\frac{df(t)}{dt} = -\big(1 - \eta(t) \times H(t)\big)\beta f(t)V(t), \quad (9)$$

$$\frac{dV(t)}{dt} = \big(1 - \eta(t) \times H(t)\big)\gamma f(t)V(t) - \delta V(t). \quad (10)$$

Here $H(t)$ is a Heaviside step function defined as $H(t) = 0$ if $t < T$: otherwise $H(t) = 1$, where T is the initiation timing of the treatment, and the anti-SARS-CoV2 effect for $t > T$ are described as

$$\varepsilon(t)\ (\text{or}\ \eta(t)) = 1 - f_u\big(D(t)\big) = 1 - \frac{1}{1 + \left(\frac{D(t)}{IC_{50}}\right)^m}, \qquad D(t) = C_{max}e^{-kt}$$

where C_{max} and k are the peak drug concentration and the elimination rate for corresponding drug, respectively. The parameter values for each drug used here are summarized in **Table S1 and S2**. Antiviral activities of CEP and NFV were calculated with the expected pharmacokinetics in human lung, based on the pharmacokinetics information for human peripheral blood and that for rat lung and peripheral blood for normalization (Yokoshima et al., 1986; Shetty et al., 1996; Ford et al., 2004).

For anti-SARS-CoV-2 therapies with double-drug combinations, we extended as the following PK/PD/VD model assuming the dose model;

$$\frac{df(t)}{dt} = -\big(1 - \eta(t) \times H(t)\big)\beta f(t)V(t), \quad (11)$$

$$\frac{dV(t)}{dt} = \big(1 - \varepsilon(t) \times H(t)\big)\big(1 - \eta(t) \times H(t)\big)\gamma f(t)V(t) - \delta V(t). \quad (12)$$

Here $H(t)$ is a Heaviside step function defined as $H(t) = 0$ if $t < T$: otherwise $H(t) = 1$, and the anti-SARS-CoV2 effect are described as

$$\varepsilon(t) = 1 - f_u^A(D_A^{\mathrm{com}}(t)\,) = 1 - \frac{1}{1 + \left(\frac{D_A^{\mathrm{com}}(t)}{IC_{50}^A}\right)^{m_A}},$$

$$\eta(t) = 1 - f_u^B\big(D_B^{\mathrm{com}}(t)\big) = 1 - \frac{1}{1 + \left(\frac{D_B^{\mathrm{com}}(t)}{IC_{50}^B}\right)^{m_B}},$$

$$D_A^{\mathrm{com}}(t) = C_{\mathrm{max}}^A e^{-k_A t}\left(1 + a_{AB}\frac{D_B^{\mathrm{com}}(t)}{IC_{50}^B + D_B^{\mathrm{com}}(t)}\right)^{-1},$$

$$D_B^{\mathrm{com}}(t) = C_{\mathrm{max}}^B e^{-k_B t}\left(1 + a_{BA}\frac{D_A^{\mathrm{com}}(t)}{IC_{50}^A + D_A^{\mathrm{com}}(t)}\right)^{-1}.$$

Note that we here evaluated the double-drug combination of NFV and CEP (**Fig. 7**), and the pharmacokinetics of NFV and CEP, $D_A^{\mathrm{com}}(t)$ and $D_B^{\mathrm{com}}(t)$, under the combination, are different from those, $D_A(t)$ and $D_B(t)$, under the single-drug treatment because of the effective drug concentration.

Evaluation of outcomes for anti-SARS-CoV-2 therapies

The antiviral effect of the anti-viral therapy on SARS-CoV-2 dynamics using Eqs. (7-12) and our estimated parameter values was calculated (**Fig. 7**). We evaluated the outcomes for the therapies defined as “period until virus elimination” and “reduction of cumulative virus production” (**Fig. S3**). Note that the cumulative virus production, i.e., the area under the curve of viral load (AUC: $\int_0^{T_D} V(s)ds$), for SARS-CoV-2 was calculated, where T_D is the time for SARS-CoV-2 achieved the detection limit.

Supplemental Figure

Fig. S1.

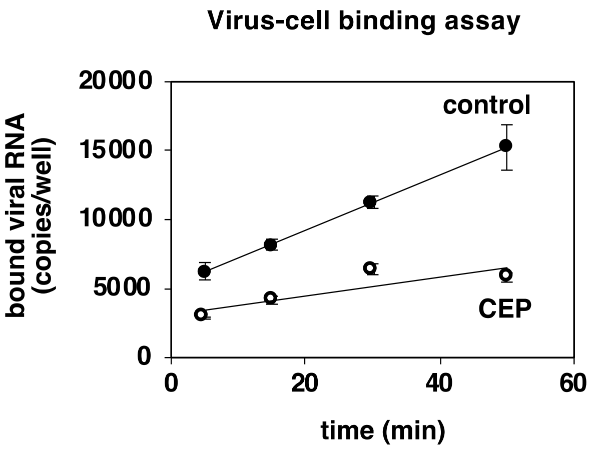

Fig. S1. SARS-CoV-2 binding to cells was inhibited under CEP treatment, related to Figure 4. SARS-CoV-2 pretreated with DMSO (control) or CEP was exposed to VeroE6/TMPRSS2 cells at an MOI of 0.001 in the presence of DMSO (control) or CEP for 5, 15, 30, and 50 min at 4˚C to allow virus-cell binding but not the following steps. After extensive wash, viral RNA was extracted from cells and was quantified by real time RT-PCR analysis. Viral RNA levels bound to cells were linearly increased along with the incubation time and CEP reduced the cell-bound viral RNA at any time points examined. This data was from three independent experiments (mean ± SD).

Fig. S2.

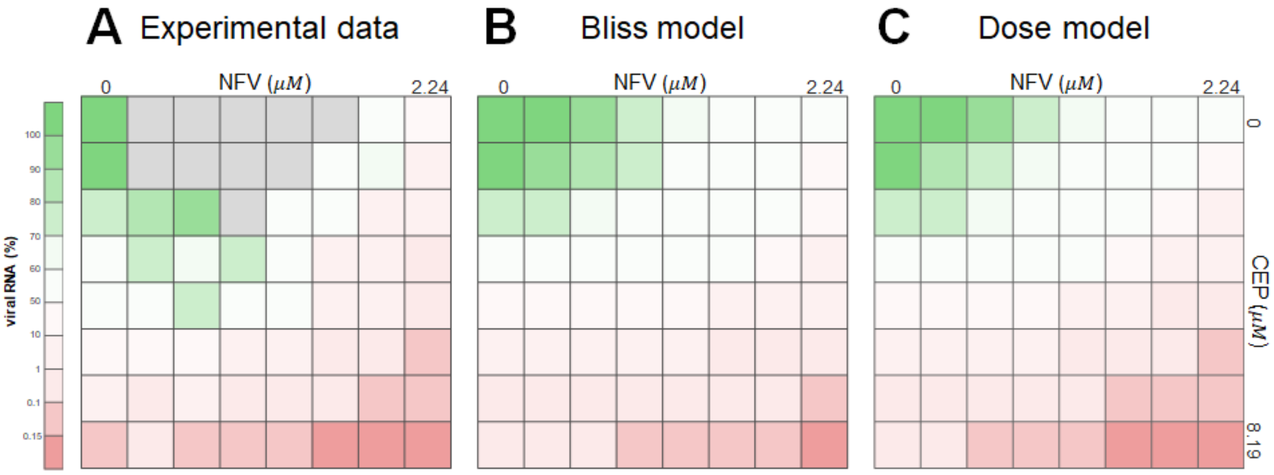

Fig. S2. Comparison of experimental data, Bliss model and Dose model for the double-drug combinations, related to Figure 6. Dose-response matrix of the double-drug combination (corresponding to **Fig. 6A**) are plotted in **(A)**, and the expected anti-SARS-CoV-2 effects of the double-drug combination (NFV and CEP) by the Bliss model and Dose model are plotted in **(B)** and **(C)**, respectively. Note that experimental measurements over 100% of viral RNA (implying large experimental variation because of small dose of antiviral drugs), colored by gray, were excluded in our analysis. The ratios of the values shown in **(A)** over those in **(B)** were calculated and are depicted in **Fig. 6C** in a 3D landscape. To increase the accuracy of evaluation, we employed 1.2-fold serial dilution of NFV from 2.24 μM and 1.6-fold serial dilution of CEP from 8.19 μM.

Fig. S3.

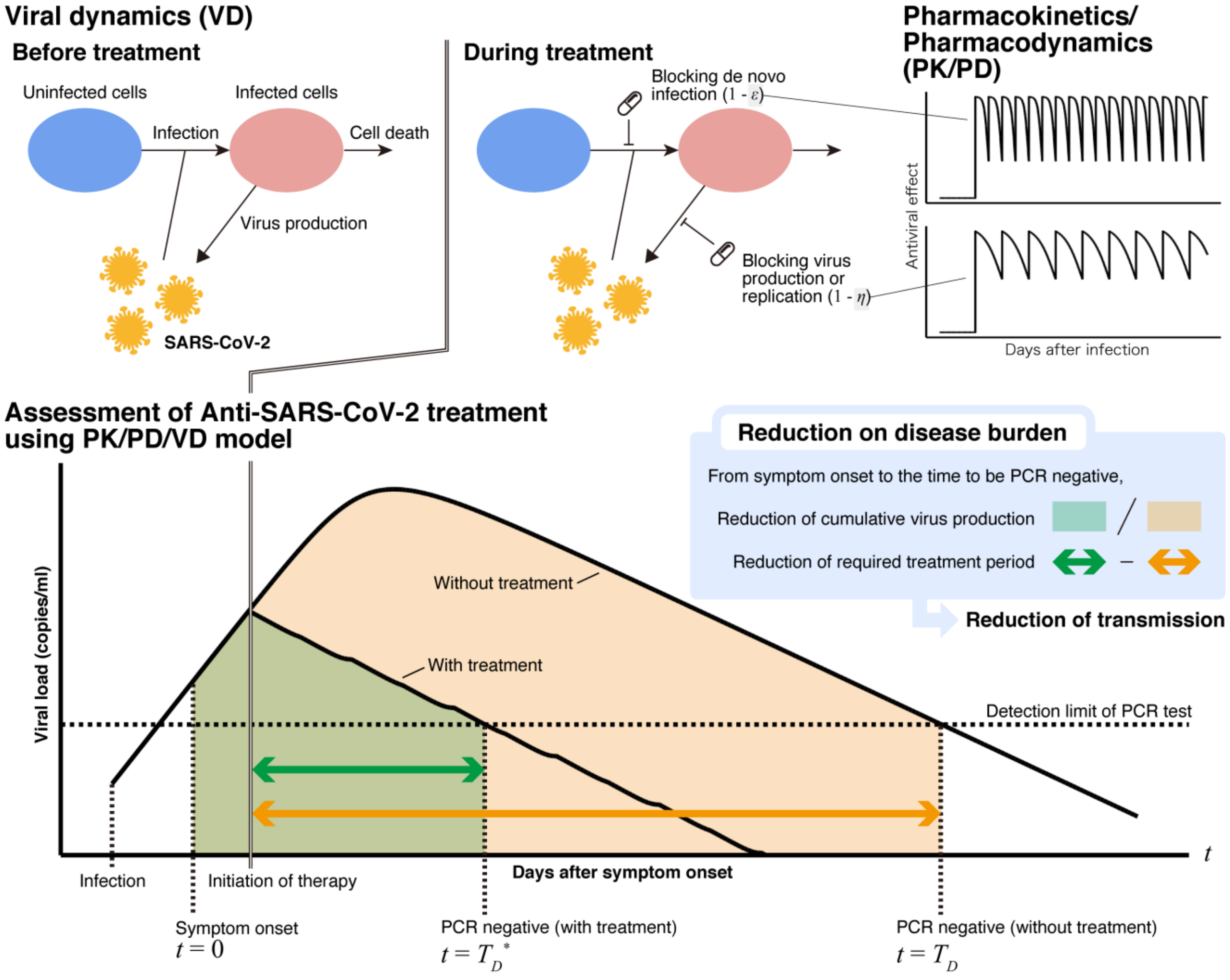

Fig. S3. Schematic representation of SARS-CoV-2 infection dynamics, related to Figure 7. A typical disease progress with viral load on patients undergoing therapy are shown. The outcomes for the therapies, that is, reduction in "period until virus elimination" and "cumulative virus production" are graphically depicted.

Supplemental Tables

Table S1. Estimated characteristic parameters of the tested antiviral drugs, related to Figure 2.

Drug (unit)	Class	IC_{50}	m
Single-drug treatment			
Lopinavir (μM)	RI	3.609	3.852
Nelfinavir (μM)	RI	0.765	5.079
Favipiravir (μM)	RI	4.057×10^{161}	5.610×10^{-3}
Remdesivir (μM)	RI	1.577	3.048
Chloroquine (μM)	EI	1.313	1.984
Cepharanthine (μM)	EI	0.351	2.307
Combination treatment			
Nelfinavir (μM)	RI	1.317	4.043
Cepharanthine (μM)	EI	0.991	3.174

RI, replication inhibitor; EI, entry inhibitor

IC_{50}, 50% inhibitory concentration

m, slope of the dose-response curve (i.e., Hill coefficient)

Table S2. Summary of pharmacokinetic parameters of anti-SARS-CoV-2 drugs, related to Figure 7.

Parameter name	Symbol	Unit	Nelfinavir*	Cepharanthine**	
				i.v.	p.o.
Single-compartment model					
Maximum concentration	C_{max}	μM	9.32	12.3	3.49×10^{-2}
Degradation rate	k	day^{-1}	4.89	0.318	
Dosing schedule					
Initiation of treatment	t^*	day		0.500	
Dosing interval	τ	day	0.333	7.00	1.00

Nelfinavir: 500 mg, TID, orally

Cepharanthine: 25mg, intravenous drip (i.v.) or 10 mg, oral administration (p.o.)

* Expected pharmacokinetics information in human lung. We estimated the scaling parameter of C_{max} between lung and peripheral blood in rats (Shetty et al., 1996). We then calculated C_{max} in human lung based on that in human peripheral blood assuming the scaling parameter is the same between humans and rats. Since the half-life of NFV in the cells was reported to be almost the same as that in plasma (Ford et al., 2004), we used the information for the half-life of NFV in plasma for that in the lung.

** Expected pharmacokinetics information in human lung. We estimated the degradation rate, k, in rat lung (Yokoshima et al., 1986), and the scaling parameter of C_{max} between lung and peripheral blood in rat. Then we used the degradation rate for human lung and calculated C_{max} in human lung by that in human peripheral blood assuming the scaling parameter is same between humans and rats.

Table S3. Estimated population parameters and initial values for SARS-CoV-2 infection, related to Figure 7.

Parameter name	Symbol	Unit	Value
Maximum rate constant for viral replication	γ	day^{-1}	3.16
Rate constant for virus infection	β	(copies/ml)$^{-1}$ day^{-1}	9.77×10^{-6}
Death rate of infected cells	δ	day^{-1}	0.615
Initial viral load	$V(0)$	copies/ml	5.64×10^{3}

Supplemental References

Bliss, C. (1939). The toxicity of poisons applied jointly1. Annals of applied biology. 26(3), 585-615.

Cao, B., Wang, Y., Wen, D., Liu, W., Wang, J., Fan, G., Ruan, L., Song, B., Cai, Y., Wei, M., et al. (2020). A Trial of Lopinavir-Ritonavir in Adults Hospitalized with Severe Covid-19. N Engl J Med. Published online 2020/03/19 DOI: 10.1056/NEJMoa2001282.

Corman, V.M., Landt, O., Kaiser, M., Molenkamp, R., Meijer, A., Chu, D.K.W., Bleicker, T., Brunink, S., Schneider, J., Schmidt, M.L., et al. (2020). Detection of 2019 novel coronavirus (2019-nCoV) by real-time RT-PCR. Euro Surveill. 25(3). Published online 2020/01/30 DOI: 10.2807/1560-7917.ES.2020.25.3.2000045.

Friesner, R.A., Banks, J.L., Murphy, R.B., Halgren, T.A., Klicic, J.J., Mainz, D.T., Repasky, M.P., Knoll, E.H., Shelley, M., Perry, J.K., et al. (2004). Glide: a new approach for rapid, accurate docking and scoring. 1. Method and assessment of docking accuracy. J Med Chem. 47(7), 1739-1749. Published online 2004/03/19 DOI: 10.1021/jm0306430.

Halgren, T.A., Murphy, R.B., Friesner, R.A., Beard, H.S., Frye, L.L., Pollard, W.T., and Banks, J.L. (2004). Glide: a new approach for rapid, accurate docking and scoring. 2. Enrichment factors in database screening. J Med Chem. 47(7), 1750-1759. Published online 2004/03/19 DOI: 10.1021/jm030644s.

Ikeda, H., Nakaoka, S., de Boer, R.J., Morita, S., Misawa, N., Koyanagi, Y., Aihara, K., Sato, K., and Iwami, S. (2016). Quantifying the effect of Vpu on the promotion of HIV-1 replication in the humanized mouse model. Retrovirology. 13, 23. Published online 2016/04/19 DOI: 10.1186/s12977-016-0252-2.

Jin, Z., Du, X., Xu, Y., Deng, Y., Liu, M., Zhao, Y., Zhang, B., Li, X., Zhang, L., Peng, C., et al. (2020). Structure of M(pro) from COVID-19 virus and discovery of its inhibitors. Nature. Published online 2020/04/10 DOI: 10.1038/s41586-020-2223-y.

Kanehisa, M., and Goto, S. (2000). KEGG: kyoto encyclopedia of genes and genomes. Nucleic Acids Res. 28(1), 27-30. Published online 1999/12/11 DOI: 10.1093/nar/28.1.27.

Kim, K.S., Ejima, K., Ito, Y., Iwanami, S., Ohashi, H., Koizumi, Y., Asai, Y., Nakaoka, S., Watashi, K., Thompson, R.N., et al. (2020). Modelling SARS-CoV-2 Dynamics: Implications for Therapy. medRxiv. 2020.2003.2023.20040493. DOI: 10.1101/2020.03.23.20040493.

Kobayashi, T., Koizumi, Y., Takeuchi, J.S., Misawa, N., Kimura, Y., Morita, S., Aihara, K., Koyanagi, Y., Iwami, S., and Sato, K. (2014). Quantification of deaminase activity-dependent and -independent restriction of HIV-1 replication mediated by APOBEC3F and APOBEC3G through experimental-mathematical investigation. J Virol. 88(10), 5881-5887. Published online 2014/03/14 DOI: 10.1128/jvi.00062-14.

Koizumi, Y., and Iwami, S. (2014). Mathematical modeling of multi-drugs therapy: a challenge for determining the optimal combinations of antiviral drugs. Theor Biol Med Model. 11, 41. Published online 2014/09/26 DOI: 10.1186/1742-4682-11-41.

Koizumi, Y., Ohashi, H., Nakajima, S., Tanaka, Y., Wakita, T., Perelson, A.S., Iwami, S., and Watashi, K. (2017). Quantifying antiviral activity optimizes drug combinations against hepatitis C virus infection. Proc Natl Acad Sci U S A. 114(8), 1922-1927. Published online 2017/02/09 DOI: 10.1073/pnas.1610197114.

Lan, J., Ge, J., Yu, J., Shan, S., Zhou, H., Fan, S., Zhang, Q., Shi, X., Wang, Q., Zhang, L., et al. (2020). Structure of the SARS-CoV-2 spike receptor-binding domain bound to the ACE2 receptor. Nature. Published online 2020/04/01 DOI: 10.1038/s41586-020-2180-5.

Matsuyama, S., Nao, N., Shirato, K., Kawase, M., Saito, S., Takayama, I., Nagata, N., Sekizuka, T., Katoh, H., Kato, F., et al. (2020). Enhanced isolation of SARS-CoV-2 by TMPRSS2-expressing cells. Proc Natl Acad Sci U S A. 117(13), 7001-7003. Published online 2020/03/14 DOI: 10.1073/pnas.2002589117.

Mizutani, T., Fukushi, S., Saijo, M., Kurane, I., and Morikawa, S. (2004). Phosphorylation of p38 MAPK and its downstream targets in SARS coronavirus-infected cells. Biochem Biophys Res Commun. 319(4), 1228-1234. Published online 2004/06/15 DOI: 10.1016/j.bbrc.2004.05.107.

Ohashi, H., Nishioka, K., Nakajima, S., Kim, S., Suzuki, R., Aizaki, H., Fukasawa, M., Kamisuki, S., Sugawara, F., Ohtani, N., et al. (2018). The aryl hydrocarbon receptor-cytochrome P450 1A1 pathway controls lipid accumulation and enhances the permissiveness for hepatitis C virus assembly. J Biol Chem. 293(51), 19559-19571. Published online 2018/11/02 DOI: 10.1074/jbc.RA118.005033.

Rut, W., Groborz, K., Zhang, L., Sun, X., Zmudzinski, M., Hilgenfeld, R., and Drag, M. (2020). Substrate specificity profiling of SARS-CoV-2 Mpro protease provides basis for anti-COVID-19 drug design. https://doi.org/10.1101/2020.03.07.981928.

Tallarida, R.J. (2001). Drug synergism: its detection and applications. J Pharmacol Exp Ther. 298(3), 865-872. Published online 2001/08/16.

Wang, M., Cao, R., Zhang, L., Yang, X., Liu, J., Xu, M., Shi, Z., Hu, Z., Zhong, W., and Xiao, G. (2020). Remdesivir and chloroquine effectively inhibit the recently emerged novel coronavirus (2019-nCoV) in vitro. Cell Res. 30(3), 269-271. Published online 2020/02/06 DOI: 10.1038/s41422-020-0282-0.

Yabuki, T., Motoda, Y., Hanada, K., Nunokawa, E., Saito, M., Seki, E., Inoue, M., Kigawa, T., and Yokoyama, S. (2007). A robust two-step PCR method of template DNA production for high-throughput cell-free protein synthesis. J Struct Funct Genomics. 8(4), 173-191. Published online 2008/01/02 DOI: 10.1007/s10969-007-9038-z.

Yokoshima, T., Tsutsumi, S., Ohtsuki, T., Takaichi, M., Nakajima, T., and Akasu, M. (1986). Studies on metabolic fate of cepharanthine: Absorption, distribution, metabolism and excretion in rats. Pharmaceutical Regulatory Science. 17(3), 458-479.

Young, B.E., Ong, S.W.X., Kalimuddin, S., Low, J.G., Tan, S.Y., Loh, J., Ng, O.T., Marimuthu, K., Ang, L.W., Mak, T.M., et al. (2020). Epidemiologic Features and Clinical Course of Patients Infected With SARS-CoV-2 in Singapore. Jama. Published online 2020/03/04 DOI: 10.1001/jama.2020.3204.

Zimmer, A., Katzir, I., Dekel, E., Mayo, A.E., and Alon, U. (2016). Prediction of multidimensional drug dose responses based on measurements of drug pairs. Proc Natl Acad Sci U S A. 113(37), 10442-10447. Published online 2016/08/27 DOI: 10.1073/pnas.1606301113.

Zou, L., Ruan, F., Huang, M., Liang, L., Huang, H., Hong, Z., Yu, J., Kang, M., Song, Y., Xia, J., et al. (2020). SARS-CoV-2 Viral Load in Upper Respiratory Specimens of Infected Patients. N Engl J Med. Published online 2020/02/20 DOI: 10.1056/NEJMc2001737.
